# Circadian Clock Proteins and Melatonin Receptors in Neurons and Glia of the *Sapajus apella* Cerebellum

**DOI:** 10.3389/fphys.2018.00005

**Published:** 2018-02-09

**Authors:** Leila M. Guissoni Campos, Alessandre Hataka, Isis Z. Vieira, Rogério L. Buchaim, Isadora F. Robalinho, Giovanna E. P. S. Arantes, Joyce S. Viégas, Henrique Bosso, Rafael M. Bravos, Luciana Pinato

**Affiliations:** ^1^São Paulo State University (UNESP), Marília, Brazil; ^2^University of Marília (UNIMAR), Marília, Brazil; ^3^São Paulo State University - UNESP, Botucatu, Brazil; ^4^University of São Paulo - USP, Bauru, Brazil

**Keywords:** biological rhythms, melatonin, clock genes, cerebellum, motor, cognition, memory, language

## Abstract

Oscillations of brain proteins in circadian rhythms are important for determining several cellular and physiological processes in anticipation of daily and seasonal environmental rhythms. In addition to the suprachiasmatic nucleus, the primary central oscillator, the cerebellum shows oscillations in gene and protein expression. The variety of local circuit rhythms that the cerebellar cortex contains influences functions such as motivational processes, regulation of feeding, food anticipation, language, and working memory. The molecular basis of the cerebellar oscillator has been demonstrated by “clock gene” expression within cells of the cerebellar layers. Genetic and epidemiological evidence suggests that disruption of circadian rhythms in humans can lead to many pathological conditions. Despite this importance, data about clock gene and protein expression in the cerebellum of diurnal (day-active) species, specifically primates, is currently poorly explored, mainly in regard to cellular identity, as well as the relationship with other molecules also involved in cerebellar functions. These studies could contribute to clarification of the possible mechanisms behind cerebellar rhythmicity. Considering that calcium binding proteins (CaBPs) play crucial roles in preserving and modulating cerebellar functions and that clock gene expression can be controlled by afferent projections or paracrine circadian signals such as the hormone melatonin, the present study aimed to describe cellular identities, distribution patterns and day/night expression changes in PER1, PER2, CaBPs, and MT_1_ and MT_2_ melatonin receptors in the cerebellar cortex of a diurnal primate using conventional fluorescence and peroxidase-antiperoxidase immunocytochemical techniques. PER1 and PER2 immunoreactive (IR) cells were observed in the Purkinje cells of the cerebellum, and MT_1_ and MT_2_ receptors were localized around Purkinje cells in the Pj layer in Bergmann cells. This identity was confirmed by the S100β-IR of these cells. The highest expression of PER seen in the daytime analysis coincided with the highest expression of melatonin receptors. CaBPs showed day/night morphological and density changes in the cerebellar cortex. The presence of the same temporal variations in the expression of PER in the Pj neurons and in MT_1_ and MT_2_ receptors in Bergmann cells indicates a possible relation between these cells during the rhythmic processing of the cerebellum, in addition to the CaBP temporal morphological and density changes.

## Introduction

Oscillations in circadian rhythms are important for the anticipation of daily and seasonal environmental rhythms. They are provided by the suprachiasmatic nucleus (SCN) of the hypothalamus, which controls the circadian rhythms of physiological, endocrine and behavioral processes (Reppert and Weaver, 2002). At the molecular level, this ability of the SCN consists of a self-sustained autoregulatory feedback mechanism reflected by the rhythmic expression of clock genes, i.e., *Per1, Per2, Per3, Cry1, Cry2, Clock, Arntl*, and *Nr1d1* (Lowrey and Takahashi, 2011).

In addition to the SCN, there are extrahypothalamic oscillators in other encephalic areas (Reppert and Weaver, 2002; Campos et al., 2015a,b) including a circadian oscillator in the cerebellar cortex, as demonstrated in rats and mice (Mendoza et al., 2010; Rath et al., 2012). Indeed, the cerebellar cortex contains a variety of local circuit rhythms, from the rhythms in the cerebellar cortex *per se* to those dictated from important afferents (Mendoza et al., 2010).

Predominantly involved in motor coordination and learning (Grimaldi and Manto, 2012; Buckner, 2013), the cerebellum has also been implicated in motivational processes (Caston et al., 1998), the regulation of feeding, food anticipation (Zhu and Wang, 2008; Mendoza et al., 2010), language (Verly et al., 2014), emotion regulation (Andreasen and Pierson, 2008), attention (Gottwald et al., 2003), and working memory (Ravizza et al., 2006).

The molecular basis of the cerebellar oscillator has been demonstrated by clock gene expression in the cerebellar cortex of rodents (Shieh, 2003; Mendoza et al., 2010; Rath et al., 2012; Paulus and Mintz, 2016). Some clock genes, i.e., *Per1, Per2, Per3, Arntl, Cry1, Nr1d1*, and *Dbp*, are rhythmically expressed (Takumi et al., 1998; Rath et al., 2012) while others, i.e., *Cry2* and *Clock*, are constitutively expressed (Guilding and Piggins, 2007; Rath et al., 2012).

Considering that all of these studies were carried out in rodents, nocturnal animals, a question remains whether clock proteins are expressed similarly in the cerebellum of primates, animals of diurnal habits. The characterized circadian gene expression of the human brain demonstrates a rhythmic increase and decrease in gene expression in regions outside of the suprachiasmatic nucleus (Li et al., 2013).

Another unresolved issue is, what determines the rhythmic expression of the clock genes in the cerebellum? Some results have shown that this expression is either independent of the SCN, as seen in an *in vitro* study (Mendoza et al., 2010), or dependent on the SCN, as seen in an *in vivo* study (Rath et al., 2012). Even in the latter case, since direct neuronal projections linking the SCN to the cerebellum have not been described, it has been assumed that this SCN influence on the cerebellum could be due to indirect cues in circadian physiology.

In addition to the possibility of cerebellar clockwork being reset by indirect cues via multiple neural projections between the hypothalamus and brainstem (Zhu and Wang, 2008), blood-borne signals could alternatively reach the cerebellar cortex. In fact, the cerebellum is known to be the target of the neuroendocrine system involved in circadian timing (El Messari et al., 1998; Choeiri et al., 2002), including the hormone melatonin. This target is marked by the presence of the G-protein coupled receptors named melatonin receptor type 1 (MT_1_) and type 2 (MT_2_) (Mazzucchelli et al., 1996; Adamah-Biassi et al., 2014; Lacoste et al., 2015; Pinato et al., 2015).

Here, we propose an investigation of cellular temporal characteristics in the cerebellar cortex of primates, by assessing the day/night expression of the PER1 and PER2 proteins, MT_1_ and MT_2_ melatonin receptors, and the calcium-binding proteins (CaBPs) calbindin (CB) and calretinin (CR). The CaBPs partially operate as buffers, decreasing the concentration of cytoplasmic Ca^2+^ in neurons, in addition to participating in enzyme activity and gene transcription (Barski et al., 2003). The choice for including CaBPs in this investigation was motivated by the fact that changes in the expression of CaBPs may be correlated with the neurochemical, morphological, and functional results seen in the cells (Babij et al., 2013). Alterations in CaBP levels in the cerebellum have been demonstrated in pathological situations (Soghomonian et al., 2017), but there are no data about possible day/night variations in primates under normal conditions. The relevance of exploring such temporal characteristics lies in the fact that these oscillations could have a definitive impact on the way information is processed in the cerebellum.

## Methods and materials

### Ethical approval

The procedures involving animal use were compliant with the National Research Council (US) Committee on Guidelines for the Use of Animals in Neuroscience and Behavioral Research (2003) and were approved by the local ethics committee (FOA/UNESP process no. 00259/2013).

### Animals

Brain slices were obtained from six adult male capuchin monkeys (*Sapajus apella*) (2–3 kg) from the Center of Tufted Capuchin Monkey Procreation of the São Paulo State University (UNESP), Araçatuba, SP, Brazil. Animals were housed in a room with a transparent and retractable roof in individual cages under natural light, temperature, and humidity conditions and fed with a controlled diet consisting of eggs, fruit, granulated protein and dried corn; water was provided *ad libitum*. In the experiments, 6 a.m. o'clock (sunrise time) was considered to be zeitgeber time 0 (ZT 0), and sunset time started at ~6 p.m. (ZT 12). Following these time parameters, animals were anesthetized and perfused at ZT10 (daytime point) and ZT 19 (nighttime point, anesthetized in dim light), with an *N* = 3 per ZT. The analyze of PER expression in the cerebellum at ZT 10 and ZT 19 in the present study, was based on the possibility of relating the results of PER2 expression in the cerebellum and results of SCN in this species which had showed a peak around ZT 9 and the nadir around ZT 18 (Rocha et al., 2014).

### Tissue collection

Following the protocol described by Campos et al. (2014), after transcardiac perfusion, the brains were cryoprotected and cryosectioned, and sections of 30 μm were stored as 10 different stepwise series in an anti-freeze solution. The coronal sections of one series, representing the entire extent of the cerebellum, were placed in rostrocaudal order. After this, five sections representing the same rostrocaudal levels were processed for each antibody.

### Immunohistochemistry

Brain sections were processed using immunohistochemical techniques for CB, CR, PER1, PER2, NeuN (neuronal marker), MT_1_ and MT_2_. The sections were washed using a solution of TBS-TX buffer (0.05 M), incubated for 48 h at 4°C in a solution containing 0.05 M TBS-TX buffer, 2% normal serum (Vector Laboratories, CA, USA) and the appropriate primary antibody: anti-CB (1:7500, Abcam, MA, USA), anti-CR (1:7500, Abcam, MA, USA), anti-PER1 (1:200, Abcam, MA, USA), anti-PER2 (1:500, Santa Cruz, TX, USA), anti-NeuN (1:1000, Abcam, MA, USA), anti-MT_1_ (1:200, Santa Cruz Biotechnology, TX, USA) or anti-MT_2_ (1:200, Santa Cruz Biotechnology, TX, USA). A combination of MT_1_ and NeuN or MT_2_ and NeuN primary antibodies was added to separate sections for 48 h. Negative staining controls were performed by omitting the primary CB and CR antibodies.

Following the protocol described in Campos et al. (2014), primary antibodies were added to separate sections for 48 h. Next, the sections with primary antibodies against PER1, PER2, NeuN, MT_1_ and MT_2_ were washed in buffer and incubated for 2 h in Alexa-488 donkey-anti-rabbit (1:200, Jackson Immunoresearch, cod. 711-545-152, PA, USA) and Cy3-labeled donkey-anti-goat (1:200, Jackson Immunoresearch, cod. 705-165-147, PA, USA) fluorescent secondary antibodies specific for each species of primary antibody. The pre-adsorption of the anti-MT_1_ and anti-MT_2_ antibodies with the immunogenic peptides MEL-1A-R (N-20) P (Santa Cruz Biotechnology, sc13179 peptide, TX, USA), and MEL-1B-R (G-20) P (Santa Cruz Biotechnology, sc28453 peptide, TX, USA) eliminated any positive staining in the cerebellum sections. Coverslips were placed on the slides using glycerol buffer as the mounting medium.

Negative staining controls were performed by adding Per1 (E-8) blocking peptide (Santa Cruz Biotechnology, sc-398890 P, TX, USA) and Per2 control/blocking peptide #1 PER2 (1-P) (Alpha Diagnostic International, Inc., TX, USA) to the primary incubation solution of PER1 and PER2 antibodies, which blocked PER1 and PER2 staining.

The sections with the primary antibodies CB and CR were washed with 0.05 M TBS-TX and incubated in a biotinylated secondary antibody specific to the primary antibody species, diluted (1:200) in the same solution as the primary, for 2 h. The sections were washed again with 0.05 M TBS-TX, incubated in a solution containing avidin-biotin complex (Vector Laboratories, CA, USA) for 2 h, and washed with Tris–HCl buffer (pH 7.6). Labeling was developed using 3,3′-diaminobenzidine tetrahydrochloride (DAB) (Sigma—Aldrich, MO, USA) as a chromogen. Next, the sections were mounted on gelatin-coated slides and dehydrated; coverslips were placed on the slides using DPX as the mounting medium (Sigma—Aldrich, MO, USA).

To ensure that sample differences would not reflect different efficiencies of immunohistochemical labeling, brain sections from the two different ZTs were processed and incubated in the same solution at the same time.

### Data analysis

The cerebellum areas were identified using brain sections stained with Nissl and the atlases “A Stereotaxic Atlas of the Brain of Cebus Monkey” (*Cebus apella*) (Manocha et al., 1968) and “The Rhesus Monkey Brain in Stereotaxic Coordinates” (Paxinos et al., 2000). For each animal, all the coronal sections of a series, representing the full extent of the cerebellum, were placed in a rostrocaudal order. After this, five sections from each animal, similar across animals (representing the same rostrocaudal level), were processed for each antibody. The sections representing different levels of the rostrocaudal extension were adjacent among antibodies. Each coronal section was analyzed under light field and epifluorescence (Olympus BX50 microscope), and the images were acquired with cellSens software (USA). Images were acquired with adequate resolution and uniform brightness, and contrast was adjusted using Adobe Photoshop CS6, which was used similarly in all images. Five images captured randomly representing different areas of the coronal slice were then analyzed, and all visible CB-IR, CR-IR, PER1-IR, PER2-IR, MT_1_-IR, and MT_2_-IR neurons of the cerebellum layers were counted in each image. The optical density (O.D) of the immunoreactivity was also quantified using the digital image processing and analysis software ImageJ (McMaster Biophotonics Facility, Canada).

### Statistical analysis

For the statistical analyses, the average cell number or O.D of the five cerebellum coronal sections of each animal was obtained. The data are expressed as the mean ± standard error of the mean of the cell number or O.D from the three monkeys perfused at the same ZT. Mann Whitney tests were used to compare the two ZTs. Values of *P* < 0.05 were considered statistically significant. As demonstrated by the standard errors of the mean, the intra-individual variability was small for the analyzed parameters.

## Results

**Figure 1:**
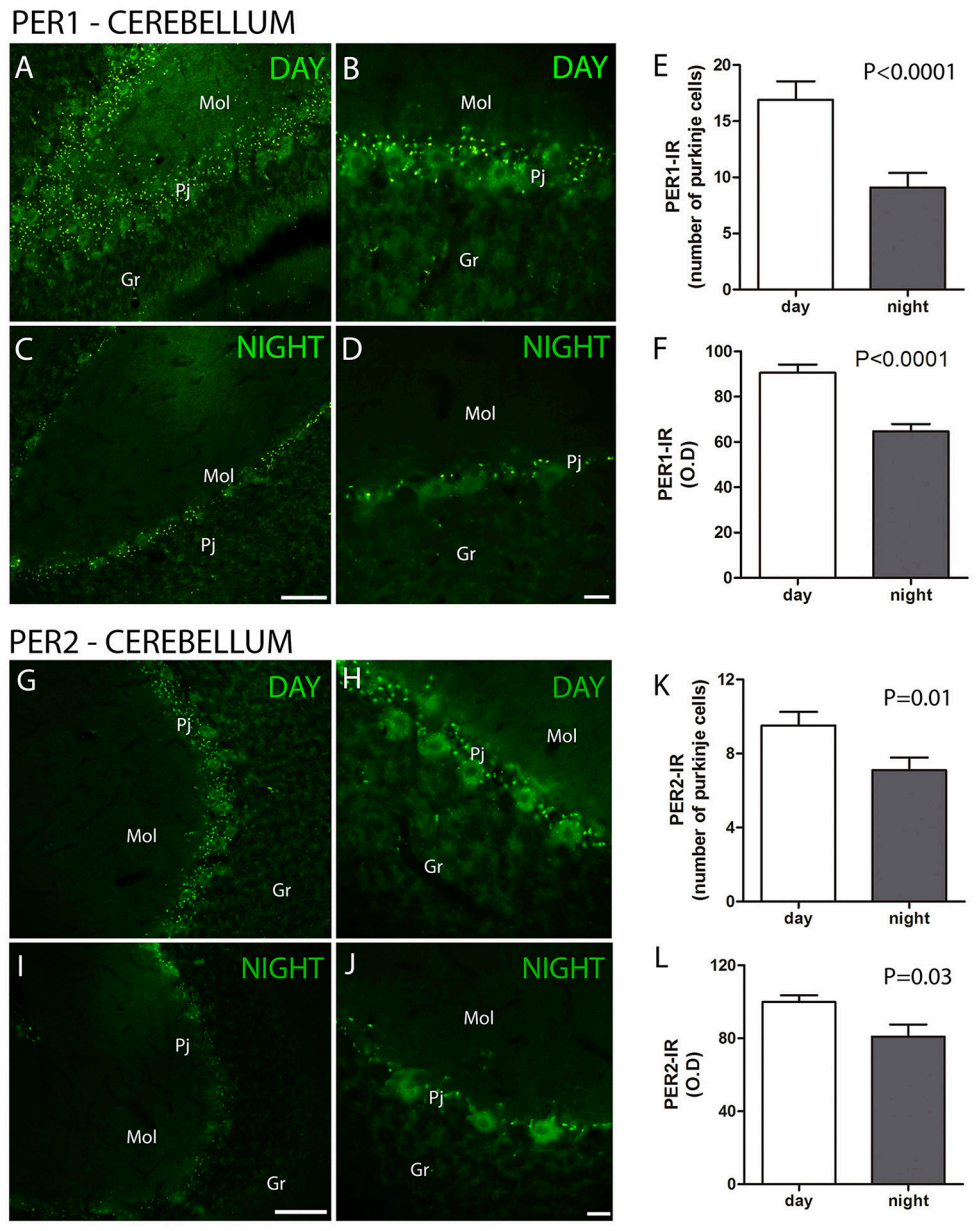
The distribution of PER1 and PER2 proteins in the cerebellar cortex in the primate *Sapajus apella*. Photomicrographs of frontal brain sections show PER1-IR cells **(A–D)** (green) or PER2-IR cells **(G–J)** at two time points, one during the day (ZT 10) and another at night **(C,D)**. The graphs show the means ± standard error of the mean of the number **(E)** and IR intensity (O.D) quantification **(F)** of PER1-IR **(E,F)** or PER2-IR **(K,L)** cells in the Purkinje cell layers of monkeys perfused at day or nighttime points. Bar = 100 or 50 μm. Purkinje cell layer (Pj); molecular layer (mol); granular layer (Gr).

The clock gene proteins PER1 and PER2 along with melatonin receptors and calcium binding proteins were analyzed in the layers of the cerebellar cortex of the primate *S. apella*. The distribution of the PER1 protein in the cerebellar cortex was detected in Purkinje cell layer (Pj) but absent in the granular (Gr) and molecular (Mol) layers. The cell-IR number (PER1, ZT 10 = 16.9 ± 1.6 vs. ZT 19 = 9.1 ± 1.3, Z = 67.5, *P* < 0.0001) and IR optical density (O.D) (PER1, ZT 10 = 90.6 ± 3.6 vs. ZT 19 = 64.7 ± 3.3, Z = 66, *P* < 0.0001) quantification indicated that PER1 expression was higher at ZT10 than in the ZT 19 (Figures 1A–F).

The distribution of the PER2 protein in the cerebellar cortex was also detected in the Pj cell layer. The cell number (PER2, ZT 10 = 9.5 ± 0.7 vs. ZT 19 = 7.1 ± 0.7, Z = 269.5, *P* = 0.01) and IR optical density (O.D) (PER2, ZT 10 = 99.9 ± 3.5 vs. ZT 19 = 80.9 ± 6.6, Z = 293, *P* = 0.03) quantification indicated that PER2 expression was higher at ZT 10 than at ZT 19, while it was absent in the Gr and Mol layers (Figures 1G–L).

**Figure 2:**
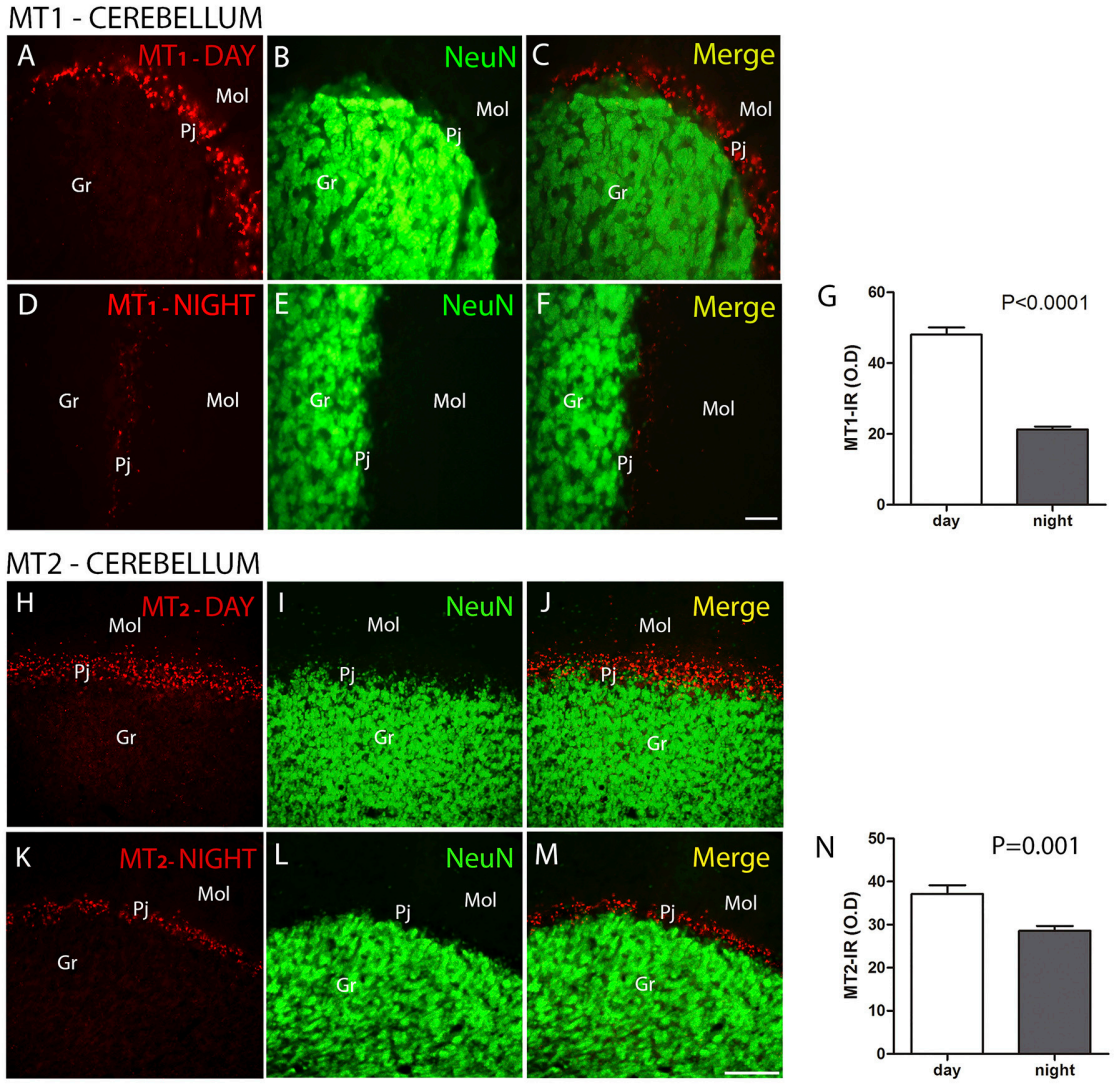
The distribution of MT_1_ and MT_2_ proteins in the cerebellar cortex in the primate *Sapajus apella*. Photomicrographs of frontal brain sections show MT_1_ protein **(A–F)** (red) or MT_2_ protein **(H–M)** (red) and NeuN cells (green) at two time points, one during the day (ZT 10) and another at night (ZT 19). The graphs show the means ± standard error of the mean of the IR intensity (O.D) quantification of MT_1_-IR cells **(G)** or MT_2_-IR cells **(N)** in the Purkinje cell layers of monkeys perfused at day or nighttime points. Bar = 50 μm. Purkinje cell layer (Pj); molecular layer (mol); granular layer (Gr).

**Figure 3:**
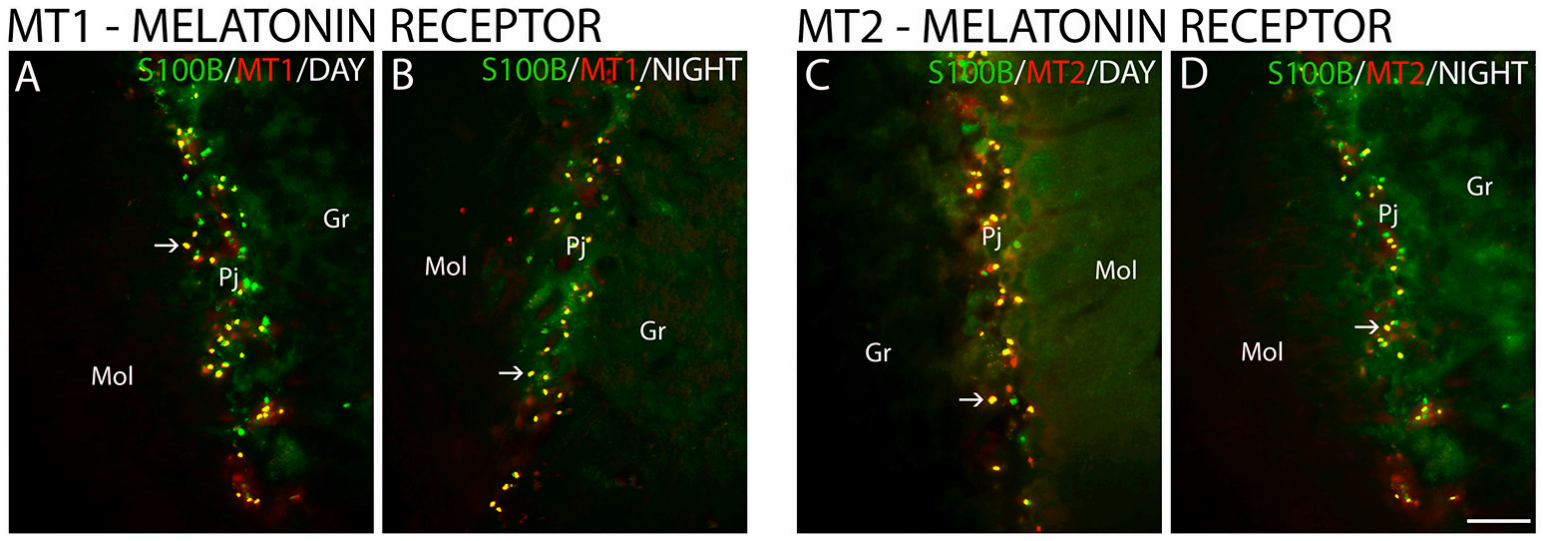
The distribution of S100β, MT_1_ and MT_2_ proteins in the cerebellar cortex in the primate *Sapajus apella*. Photomicrographs of frontal brain sections show MT_1_ protein **(A,B)** (red) or MT_2_ protein **(C,D)** (red) and S100β (green) at two time points, one during the day (ZT 10) and another at night (ZT 19). The arrows indicate the co-localization (merge) of S100β and melatonin receptors. Bar = 100 μm.

**Figure 4:**
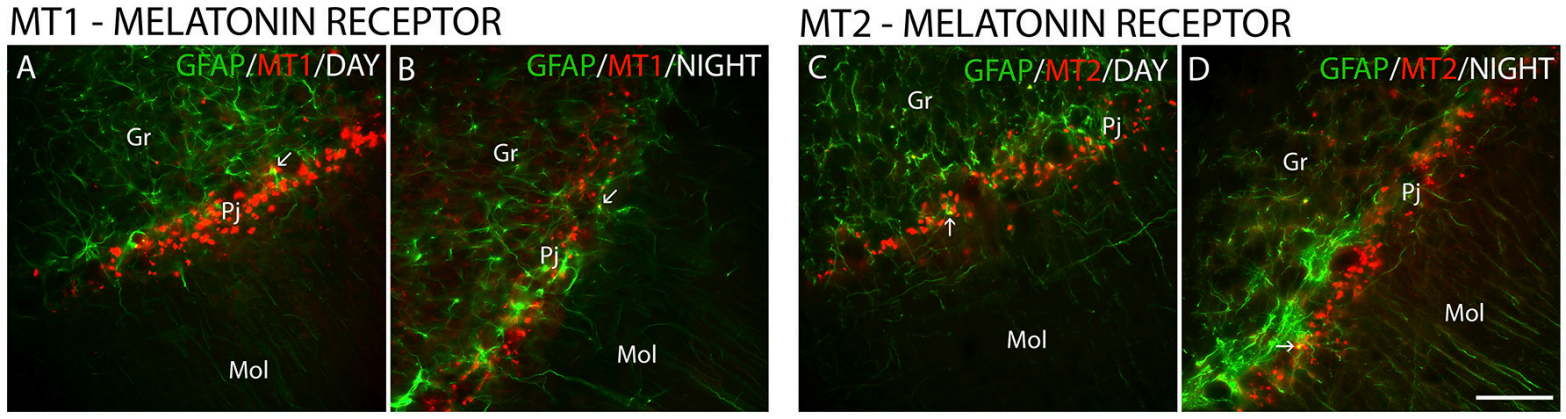
The distribution of GFAP, MT_1_ and MT_2_ proteins in the cerebellar cortex in the primate *Sapajus apella*. Photomicrographs of frontal brain sections show MT_1_ protein **(A,B)** (red) or MT_2_ protein **(C,D)** (red) and GFAP (green) at two time points, one during the day (ZT 10) and another at night (ZT 19). The arrows indicate the co-localization (merge) of GFAP and melatonin receptors. Bar = 100 μm.

The MT_1_ and MT_2_ receptors showed differences in IR intensity (O.D) between the day- and nighttime points, as well as specific patterns of distribution between the layers analyzed. The immunofluorescence analysis revealed a prevalence of both MT_1_-IR (MT1, ZT 10 = 48.0 ± 2.1 vs. ZT 19 = 21.2 ± 0.8, Z = 6.0, *P* < 0.0001) (Figures 2A–G) and MT_2_-IR (MT2, ZT 10 = 37.1 ± 2.0 vs. ZT 19 = 28.5 ± 1.1, Z = 149.5, *P* < 0.0001) (Figures 2H–N) at the daytime point. The higher intensity in the Pj layer and the lower intensity of MT_1_ and MT_2_ receptor expression in the Gr and Mol layers at the same coronal level of the cerebellum were seen at both the day- and nighttime points (Figure 2). Regarding the identity of the cells that showed MT_1_-IR and MT_2_-IR, the melatonin receptors were mainly localized around Purkinje cells in Bergmann cells, identified by S100β (Figure 3), and a few astrocytes (GFAP-IR) showed co-localization with these receptors (Figure 4).

**Figure 5:**
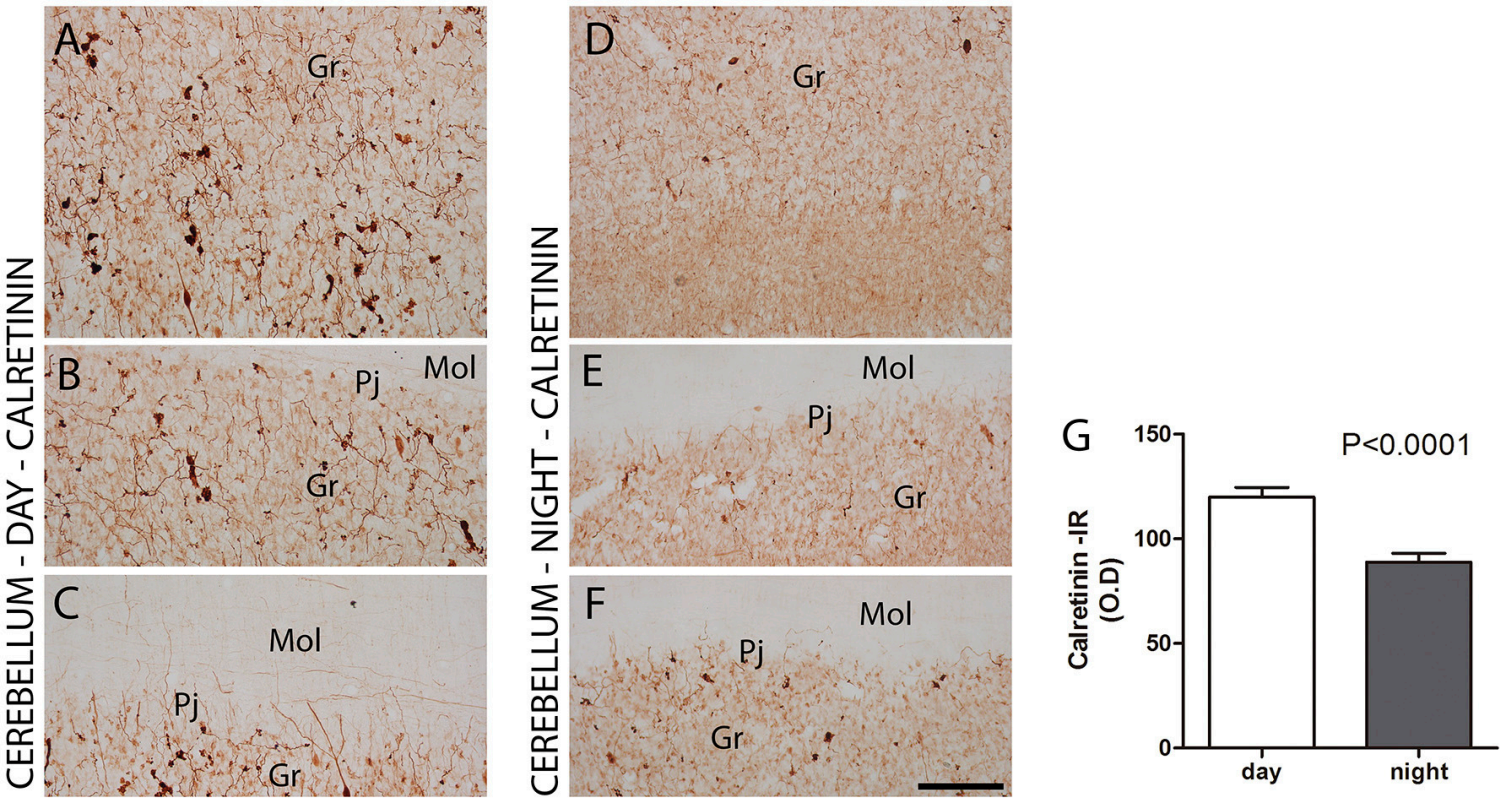
The distribution of calretinin (CR) protein in the cerebellar cortex in the primate *Sapajus apella*. Photomicrographs of frontal brain sections show CR-IR with significant expression in the granular layer **(A–F)** at two time points, one during the day (ZT 10) **(A–C)** and another at night (ZT 19) **(D–F)**. The CR-IR showed day/night variations in IR intensity (O.D) in the cells in the granular layer **(G)**. Bar = 100 μm. Purkinje cell layer (Pj); molecular layer (mol); granular layer (Gr).

CR showed differences between the day and nighttime points in IR intensity (O.D) (CR, ZT 10 = 119.9 ± 4.6 vs. ZT 19 = 88.8 ± 4.3, Z = 213, *P* < 0.0001) (Figures 5A–G) and in cell morphology. The most significant expression of CR was in the Gr layer, where these neurons showed higher expression at the daytime point than at the nighttime point (Figures 5A–G). At the daytime point, the cell bodies of CR-IR neurons were more strongly stained. In addition to the increase in density of CB-IR, these neurons showed morphological differences when comparing the two time points, with a broader dendritic arborization at the daytime point (Figures 5A–C) than at the nighttime point (Figures 5D–F).

**Figure 6:**
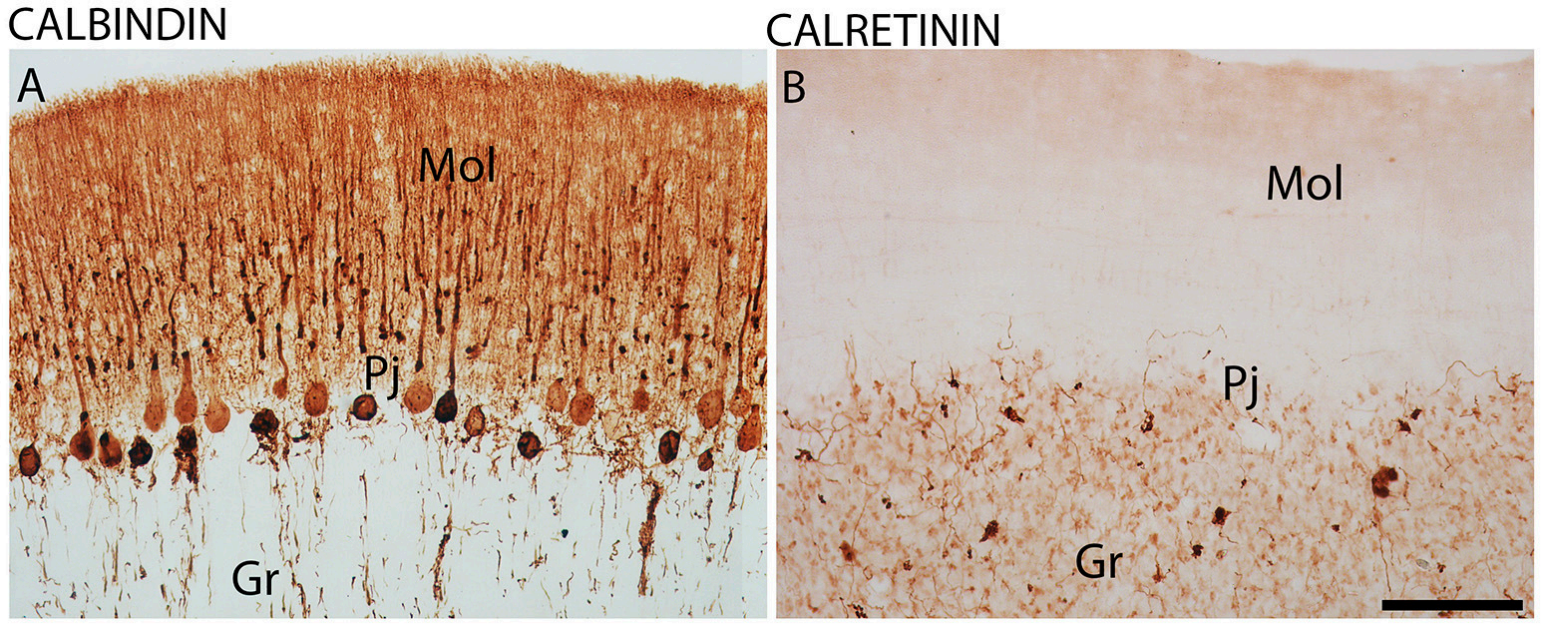
The distribution of calbindin (CB) and calretinin (CR) proteins in the cerebellar cortex in the primate *Sapajus apella*. Photomicrographs of frontal brain sections show strongly stained CB-IR cells in the Purkinje cell layer (Pj) **(A)** and CR-IR with significant expression in the granular layer (Gr) **(B)**. Bar = 100 μm. Molecular layer (mol).

**Figure 7:**
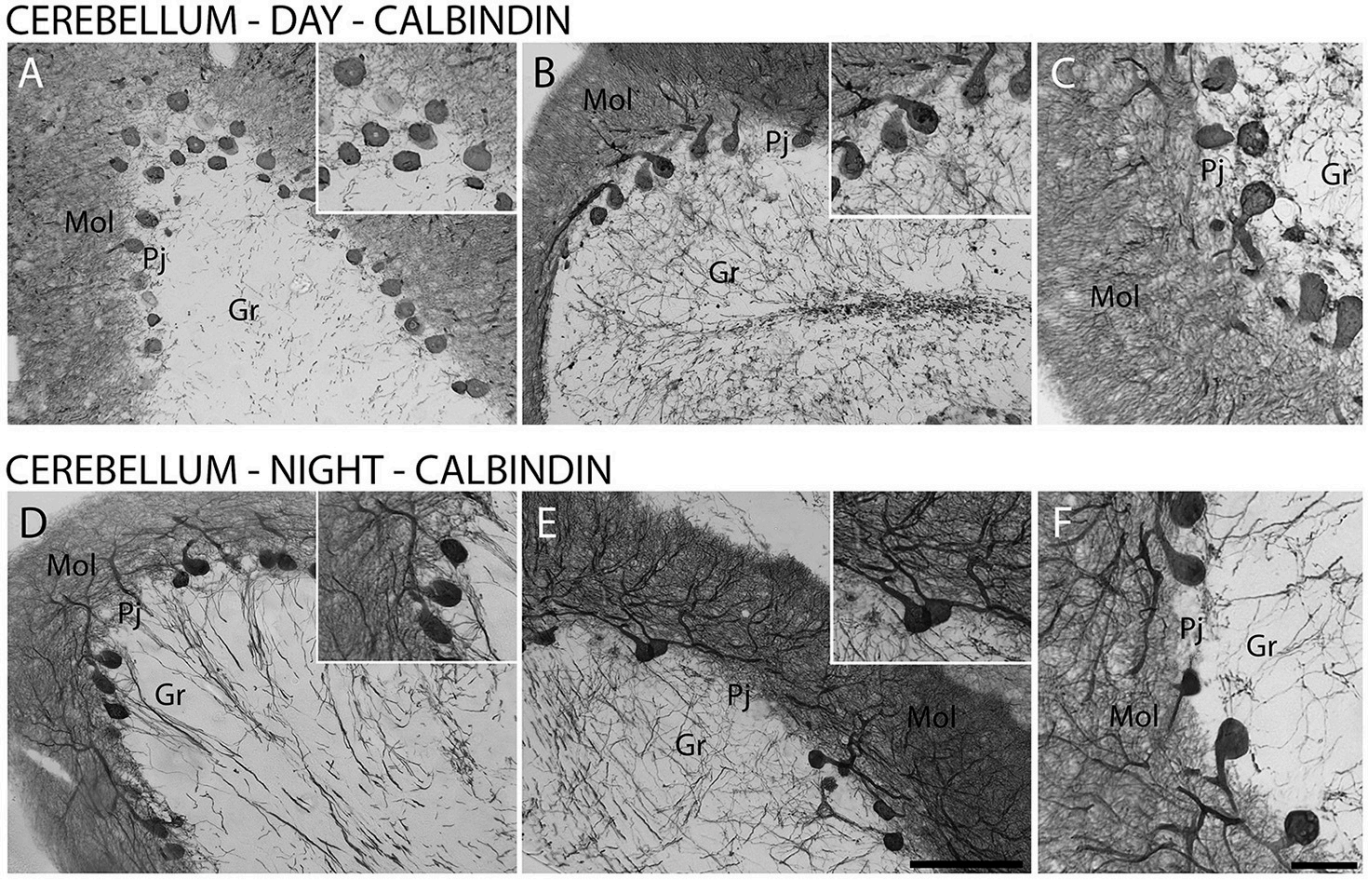
The distribution of calbindin (CB) protein in the cerebellar cortex in the primate *Sapajus apella*. Photomicrographs of frontal brain sections show strongly stained CB-IR cells in Purkinje cell layer (Pj) **(A–F)** at two time points, one during the day (ZT 10) **(A–C)** and another at night (ZT 19) **(D–F)**. The CB-IR showed day/night variations in cell morphology in the Purkinje cell layer **(D–F)**. Bar = 100 or 50 μm. Molecular layer (mol); granular layer (Gr).

Furthermore, CB-IR and CR-IR proteins showed specific patterns of distribution between the layers analyzed (Figure 6). The Purkinje cells showed strong CB-IR. At both time points, they exhibited immunoreactivity in form of fine granular deposits dispersed in the cytoplasm, showing various degrees of labeling intensity (Figure 6A). At the daytime point, immunoreactivity was observed in the bodies and proximal segments of their main processes (Figures 7A–C). At the night point, these neurons showed differences in morphology, with long fibers that reached the Mol layer of the cerebellar cortex, forming a broader dendritic arborization and extension of Purkinje cell axon collateral plexus (Figures 7D–F).

## Discussion

To determine if molecules associated with generation or modulation of biological rhythms and plasticity are expressed in the cerebellar cortex of diurnal animals, here we describe the pattern of expression of PER1 and PER2 proteins, melatonin receptors and CaBPs at two different time points in the cerebellar cortex of the primate *S. apella*. First, we demonstrated evidence that PER1 and PER2 are expressed in the Purkinje cell layer but are absent from the Gr and Mol layers of the cerebellar cortex of this primate and that these expressions exhibit a difference between the day- and nighttime points analyzed. This includes the cerebellar cortex up to the cerebral regions in which clock gene proteins variations have been detected in primates (Courtemanche et al., 2013; Rocha et al., 2014; Campos et al., 2015a,b). The pattern of distribution of PER2 protein found in the present study is in agreement with the expression of the PER2 protein in the Pj of the cerebellar cortex in rats (Mendoza et al., 2010). Similarly, the clock genes *Per1* and *Per2* are also described in the perikarya of the Pj of rodents, but different from the results of protein expression, the genes were also found in the Gr layer of rodents using hybridization techniques (Shieh, 2003; Rath et al., 2012).

In the present study, the expression of PER1 and PER2 in the cerebellar cortex was higher at the day (ZT 10) than at the night (ZT 19) time point analyzed. Previous studies in rats and mice had demonstrated a peak of PER1 and PER2 proteins or *Per1* and *Per2* gene expression at night (Akiyama et al., 2001; Farnell et al., 2008; Mendoza et al., 2010). Such differences may be related to the differences in the activity habits of the animals since the cerebellum is the major center of motor activity modulation (Doya, 2000). They could also be related to the differences in the availability of food among these animals. The cerebellum participates in the behavior of anticipating the time of food accessibility, which involves increased activity, behavioral arousal, and body temperature changes (Mistlberger, 1994; Mendoza et al., 2010). Thereby, the present study expands current knowledge on the rhythmic characteristics of the cerebellum, which was previously based on investigations showing clock gene expression in rodent and human studies (Shieh, 2003; Farnell et al., 2008; Mendoza et al., 2010; Rath et al., 2012; Li et al., 2013; Paulus and Mintz, 2016). These rhythmic clock gene and protein expressions are transduced into a rhythmic neuronal output signal that can influence other brain targets and play a crucial role in the anticipation of 24 h predictable environmental changes (Courtemanche et al., 2013). Once processed by the cerebellar cortex, temporal messages can be transferred via identified neural pathways to structures including the diencephalic and cortical areas of the sensorimotor system (Cavdar et al., 2001; Schnitzler and Gross, 2005; Courtemanche et al., 2013).

The origin of the rhythmicity of clock genes and proteins in the cerebellum has not been clarified. The molecular clockwork of the cerebellum could be autonomous or, to some extent, controlled by the master clock of the SCN (Mendoza et al., 2010; Rath et al., 2012). To explore whether or not the differences between the day- and nighttimes levels of PER 1 and PER2 proteins found in the *S. apella* are dependent on the SCN, it will be necessary (in future studies with more time points) to keep these animals in a free-running rhythm, or even to perform SCN lesions. At this time, we can only compare the results of the present study with previous results of the same species, where the PER2 protein was found also in the SCN during the day (Rocha et al., 2014), similar to the peak found in the cerebellum in the present study.

Although the mechanism responsible for conferring information on circadian time from the SCN to the cerebellar cortex remains enigmatic, external cues such as feeding schedules, neurotransmitters, and neurohormones have been shown to entrain extrahypothalamic oscillators in the brain (Verwey and Amir, 2009). One molecule that is implicated in circadian functions, relaying information about the environmental state, showing a circadian rhythm of its level, and acting directly on the master clock in the SCN is the pineal hormone melatonin. Once secreted by the pineal gland at night, melatonin enters into the blood circulatory system, through which it travels to act on different regions of the body to achieve desirable physiological responses (Costa et al., 1995; Gilgun-Sherki et al., 2001). Its influence occurs by binding to melatonin receptors, whose high density indicates that an area is a target for melatonin action (Dubocovich et al., 2003).

In the present study, MT_1_-IR and MT_2_-IR cells were mainly localized in Bergmann cells, with a higher intensity at the daytime point. Few GFAP-IR cells were colocalized with melatonin receptors independent of the time point. mRNA expression has been shown already in the human cerebellum, but without temporal information. MT_1_ mRNA was expressed in granule cells and basket-stellate cells, whereas melatonin MT_2_ mRNA was observed in Bergmann cells and astrocytes (Mazzucchelli et al., 1996; Al-Ghoul et al., 1998). MT_1_ and MT_2_ were both previously described in the cerebellum of mice (Adamah-Biassi et al., 2014) and rats (Lacoste et al., 2015). The interactions expected in this case are with melatonin acting in glial cells by modulating glutamate functions through the activation of melatonin receptors. In the cerebellum, Bergmann glial function is associated with synapses in the molecular cell layer. In the case of glutamate synapses, glial cells are thought to be involved in the re-uptake of glutamate (Ottersen et al., 1997; Miyazaki et al., 2017). Furthermore, there is evidence implicating glial cells in circadian rhythm and melatonin—related functions (Welsh and Reppert, 1996; Adachi et al., 2002). In this context, it is noteworthy that the pineal hormone melatonin, as a component of the circadian timing system, may permanently modulate circadian properties of the cerebellum through altered expression or temporal configurations of its molecular components. The possible actions of melatonin in the cerebellum could be in sensorimotor performances (Fraschini et al., 1999; Ng et al., 2017) or in neuroprotective effects (Manda et al., 2008; Pinato et al., 2015). Melatonin receptors can also be involved in the modulation of clock gene expression in the cerebellum as demonstrated in other brain areas (Dardente et al., 2003; Coelho et al., 2015; Vriend and Reiter, 2015). This result could represent an indirect pathway for the transmission of temporal cues to the cerebellar cortex.

The investigation of CR-IR and CB-IR showed cellular temporal characteristics dependent on the period of activity and rest. The subpopulations of Pj cells that appeared as strongly stained CB-IR neurons agrees with previous results in humans (Babij et al., 2013). At the nighttime point, these neurons showed morphology with longer fibers than at the daytime point, which reached the Mol layer of the cerebellar cortex. On the other hand, the most significant expression of CR was in the Gr layer, which has already been described (Ito, 1984). The present study adds the information that CR-IR neurons showed higher expression during the day than in the nighttime period, with morphological differences of dendritic arborization in the Gr layer. In other brain areas, morphological and density changes in CaBPs have also been observed (Gall et al., 2003; Campos et al., 2015a,b), i.e., in the hippocampus, which, along with the cerebellum, exercises different functions dependent on the time of day (Squire, 2007). Since the functions of CaBPs include the regulation of intracellular processes such as neuronal excitability and release of neurotransmitters (Hof et al., 1999; Schwaller et al., 2002), these patterns may represent responses to different conditions, in this case, day- and nighttime periods.

In conclusion, our data establish several characteristics that can affect the way information is processed in the cerebellum. Day/night changes in the clock proteins PER1 and PER2 are present in the Purkinje cells of the cerebellum of the primate *S. apella*. MT_1_ and MT_2_ receptors are localized mainly in Bergmann cells in the Pj layer. The highest expression of PER proteins at the daytime point coincides with the highest expression of melatonin receptors. The calcium-binding proteins showed morphological and density changes in the cerebellar cortex between the two time points analyzed.

## Author contributions

LMGC: conception or design of the work; analysis, interpretation of data for the work; drafting the work; AH: interpretation of data for the work, analysis, drafting the work; IZV: acquisition, analysis, interpretation of data for the work; drafting the work. RLB: acquisition, analysis, interpretation of data for the work; IFR: acquisition, analysis, interpretation of data for the work; drafting the work; GEPSA: acquisition, analysis, interpretation of data for the work; drafting the work; JSV: acquisition, analysis, interpretation of data for the work; drafting the work; HB: acquisition, analysis, interpretation of data for the work; drafting the work; RMB: acquisition, analysis, interpretation of data for the work; drafting the work; LP: conception or design of the work; analysis, interpretation of data for the work; drafting the work.

### Conflict of interest statement

The authors declare that the research was conducted in the absence of any commercial or financial relationships that could be construed as a potential conflict of interest.
